# Standard RTS criteria effectiveness verification using FMS, Y-balance and TJA in footballers following ACL reconstruction and mild lower limb injuries

**DOI:** 10.1038/s41598-021-81152-4

**Published:** 2021-01-15

**Authors:** Łukasz Oleksy, Anna Mika, Iwona Sulowska-Daszyk, Daniel Szymczyk, Maciej Kuchciak, Artur Stolarczyk, Radosław Rojek, Renata Kielnar

**Affiliations:** 1 Orthopaedic and Rehabilitation Department, Medical University of Warsaw, Warsaw, Poland; 2 Oleksy Medical and Sports Sciences, Łańcut, Poland; 3 Institute of Clinical Rehabilitation, University of Physical Education in Kraków, Al. Jana Pawła II 78, 31-571 Kraków, Poland; 4 Institute of Health Sciences, Medical College of Rzeszów University, Rzeszów, Poland; 5 Institute of Physical Culture Sciences, Rzeszów University, Rzeszów, Poland; 6 Physiotherapy Clinic ReSport, Tarnów, Poland

**Keywords:** Health care, Medical research, Risk factors

## Abstract

The anterior cruciate ligament (ACL) is the most frequently injured knee ligament. In previous studies, it was demonstrated that patients following ACL reconstruction may present motor deficits which may be related to increased risk of injury. The objective of the study was to determine whether players who have passed RTS assessment still have deficits in movement patterns or in neuromuscular control after such a serious injury as ACL rupture and reconstruction,. Sixty-five male football players (age 18–25 years), recruited from regional teams, were divided into three groups: (1) ACL group-subjects after ACL rupture and reconstruction (n = 24); (2) Mild Injury (MI) group-subjects after mild lower limb injuries (n = 21); and (3) Control (C) group-subjects without injuries (n = 20). For all groups, the Y-balance test, Functional Movement Screen (FMS) and Tuck Jump Assessment (TJA) were performed. For the Y-balance test in ACL group for the injured leg, significantly lower values were demonstrated for anterior reach ((ACL) 69.2 ± 5.7% vs. (MI) 74.8 ± 4.5% vs. (C) 74.0 ± 5.6%), at posterior-lateral reach ((ACL) 103.2 ± 6.4% vs. (C) 108.5 ± 6.0%) and composite score ((ACL) 93.9 ± 4.4% vs. (MI) 97.9 ± 4.3%) in comparison to the remaining two groups. In the FMS test, the ACL group had significantly lower composite score (12 ± 4 points) compared to the C group (15 ± 2 points). Also, compared to the remaining groups, subjects following ACL reconstruction demonstrated significantly lower performance in the TJA test related to the following elements: thighs do not reach parallel, thighs not equal side-to-side, foot placement not shoulder width apart, technique declines prior to 10 s and lower extremity valgus at landing. The authors have observed that athletes after ACL reconstruction still have deficits in movement patterns or in neuromuscular control despite passing the RTS and being cleared to perform sports. Creating a set of sufficiently sensitive assessment methods may significantly reduce the potential risk of injury due to the fact that diagnosed and monitored deficits may be treated on an ongoing basis. The authors suggest that individual elements of the Y-balance and TJA tests may be suitable for such specific assessment.

## Introduction

The anterior cruciate ligament (ACL) plays an important role in maintaining stability of the knee and is the most frequently injured knee ligament^1,2^. Successful ACL reconstruction is determined by many factors such as surgical technique, graft choice, graft fixation, postoperative rehabilitation and patient education^3^. Unfortunately, graft failure and contralateral ACL rupture can still occur even after successful ACL reconstruction^3,4^. The return to sport rate among elite athletes following ACL reconstruction reached 83%, but 5.2% had graft rupture^5^. Athletic performance among elite athletes who have returned to sport after ACL reconstruction often deteriorated compared to pre-injury levels and, moreover, they returned to level I sports and experienced an increase the re-injury risk four-fold compared to athletes from lower levels^6^.

The causes of ACL re-injury are not clear, but deficits in neuromuscular factors, present following injuries, have been reported^4,7^. An important issue is that many athletes who have been cleared for a return to sports (RTS) after injury still show impairment in motor control and coordination^8^. After ACL reconstruction, alterations were observed in frontal-plane knee motion during landing, sagittal plane knee moment asymmetries at initial contact, at uninvolved hip rotation net moment during landing as well as deficits regarding postural stability in the reconstructed leg^9^. These undetected deficits are suggested to be strongly associated with the rapid occurrence of compensation and pathological movement patterns, leading to further tissues overloads and often to ACL re-injury^10,11^.

Functional assessment is inexpensive and easy to use^8^. The Functional Movement Screen (FMS) and Y-balance test (YBT) are popular screening tools, which may be applied for injury risk assessment based on abnormal movement patterns, asymmetry and dynamic balance^8,14–16^. The FMS is used to assess functional movement patterns, which require both mobility and stability, helping to identify painful patterns and movement impairments^8,17,18^. The YBT is implemented to evaluate impairments of balance and dynamic control which may be associated with an increased risk of lower limb injury^8,19,20^. The Tuck Jump Assessment (TJA) test comprises a plyometric double-leg jump task evaluating side-to-side asymmetries, neuromuscular imbalances and jumping and landing technique flaws pertaining to risk of ACL injury^21–23^.

In some reports, it has been suggested that the FMS may be an effective tool during RTS evaluation^14^, but mostly for subjects without a history of ACL reconstruction^24–26^, while in others, its limited usage has been implied^27,28^. On the other hand, it has been reported that the FMS can be used to identify collegiate female athletes who are at an increased risk of sustaining a non-contact ACL or lower extremity injury^29^. Some researchers, examining the connection between dynamic balance performance and injury risk, have indicated the usefulness of YBT^16,30,31^, while others have not found any differences in YBT scores among athletes following ACL reconstruction who were (or not) cleared for return to unrestricted activity^8^. Some researchers also recommend this test in RTS^13,16,20,32,33^. Furthermore, the TJA is noted as a reliable tool in RTS evaluation among elite football players^21,22,34–37^.

Finally, because motor deficits are noted not only after such serious injury as ACL rupture, but also following mild injuries, researchers have suggested that each trauma has some consequences on the motor system^6,8,9,19,20,22,38^. It is not clear how large these deficits are after various injuries and whether passing the RTS after ACL reconstruction guarantees that they are minimal or comparable to those after mild injuries of the lower limbs common in sport.

The objective of this study was to comprehensively assess impairments in functional movement patterns, neuromuscular imbalances and deficiencies in dynamic control as well as side-to-side asymmetries in football players after ACL reconstruction and to compare them with football players suffering from mild lower limb injuries as well as those without injuries. The purpose of the present study also was to determine whether players who have passed RTS assessment still have deficits in movement patterns or in neuromuscular control after such serious injury as ACL rupture and reconstruction.

## Methods

### Participants

Sixty-five male football players recruited from regional teams participated in this study (Table 1). All subjects after ACL reconstruction previously passed the RTS which included: standard orthopedic tests, manual tests performed by a physiotherapist, muscle strength evaluation and hop tests.

**Table 1:** Subjects characteristics.

	ACL group	MI group	C group
Number of subjects (n)	24	21	20
Age	22.7 ± 3.6	20.5 ± 3.7	23.1 ± 2.8
Weight (kg)	77.3 ± 7.6	74.3 ± 9.1	75.8 ± 8.8
Height (cm)	175 ± 4	177 ± 6	178 ± 6

No significant difference was found for any variable.

Inclusion criteria:

- regular football training at regional team level;
- first and unilateral ACL rupture and reconstruction 2–3 years before the study;
- passing RTS;
- no additional injuries to contralateral leg (uninjured leg);

Exclusion criteria:

- bilateral ACL rupture and reconstruction;
- ACL graft rupture;
- ACL rupture without reconstruction;
- serious injury to contralateral leg;
- pain and movement restriction which may significantly restrict performance of tests;
- no consent to participate in research.

The participants were divided into three groups:

- ACL group (n = 24)—subjects after ACL rupture and reconstruction during previous 2–3 years who passed the RTS and were cleared to play. In this group, both lower limbs are evaluated (involved leg—after ACL reconstruction, uninvolved leg—contralateral limb without ACL injury);
- MI group (n = 21)—subjects after mild lower limb injury (grade 1 muscle strains) during previous 2–3 years (involved leg—after mild injury, uninvolved leg—contralateral limb without injury);
- C group (n = 20)—control group without injuries (the left limb was the equivalent of the involved limb while the right limb was the equivalent of the uninvolved one).

The study participants were informed in detail about the research protocol and gave their written informed consent to participate in the study. Informed consent was obtained from parents for participants under the age of 18. Approval of the Ethical Committee of Regional Medical Chamber in Kraków was obtained for this study (16/KBL/OIL/2016). All procedures were performed in accordance with the 1964 Declaration of Helsinki and its later amendments.

### Procedures

The subjects did not perform any vigorous training the day before measurements to avoid the effects of cumulative muscular fatigue. All subjects completed functional testing, consisting of the FMS test, the Y-balance test and Tuck Jump Assessment. All tests were performed by an experienced researcher who was blinded to subject group allocation. Each athlete, prior to testing, completed a 5-min warm-up. Additionally, each subject performed testing trials of each test to become fully familiar with the nature of the measurements. There were 15-min intervals between the FMS, Y-balance and TJA tests.

### Functional movement screen test

The FMS test (Functional Movement Systems Inc., Chatham, VA) includes assessment of stability and mobility within the kinetic chain of full body movements, identification of body asymmetries and recognition of overall poor quality movement patterns^39–41^. The FMS test comprised 7 tasks:

1. Deep squat;
2. Hurdle step;
3. In-line lunge;
4. Shoulder mobility;
5. Active straight leg raise;
6. Trunk stability push-up;
7. Rotary stability.

During the test, each participant was observed from the front, back and side. In asymmetric tests, both the right and left sides were assessed and the lower result was considered for the final score. Each task was performed 3 times and was scored on a scale of 0 to 3 points (3 points—movement performed correctly without compensation; 2 points—movement performed with compensation; 1 point—inability to execute the task; 0 points—pain experienced during test performance). The maximal composite score was 21 points. FMS test reliability for the ICC inter-rater ranged from 0.87 to 0.89 and for the ICC intra-rater, the range was from 0.81 to 0.91^42,43^.

### Y-balance test

The Y-balance test (YBT; Move2Perform, Evansville, IN) was performed according to criteria described by Plisky et al.^19,44^. Subjects were instructed to stand barefoot on the YBT Test Kit with one foot positioned behind the red indicator line. Next, the subject reached towards one of three directions (anterior, posterior-medial and posterior-lateral) using their non-weight-bearing lower limb to slide the moveable platform. Three reach trials were performed in each direction, first standing on the uninvolved leg and then on the one involved (on the right leg and then on the left in control group)^44^. The final score was the mean value from three trials for each leg (evaluated leg was that weight-bearing). YBT reach scores were normalized as a percentage of limb length. Limb length was measured with a tape in centimeters from the anterior–superior iliac spine to the distal aspect of the medial malleolus^19^. The composite score (CS) was calculated according to the following formula:

$${\text{CS}} = [{\text{(anterior}}\;{\text{reach}}\;{\text{distance}} + {\text{posterior - medial}}\;{\text{reach}}\;{\text{distance}} + {\text{posterior - lateral reach distance)/3}} \times {\text{leg length}}] \times {1}00\%$$

The reported reliability of the YBT was 0.85–0.91 for the ICC intra-rater and 0.85–0.93 for ICC inter-rater^44,45^.

### Tuck jump assessment test

The TJA was performed according to previously described protocols^23,46^. Subjects were asked to begin the test with feet separated at shoulder width, initiating the jump with a slight downward crouch. Then they were requested to jump repeatedly for 10 s, bringing the knees to the chest during each jump, with the thighs parallel to the floor, landing softly on the same footprint and immediately jumping again^23^. Jumping efforts were recorded with a resolution of 736 X 352 and 125 fps frame rate using the NiNOX 125 camcorder (NiNOX 125, Noraxon USA) from sagittal and frontal plane view. After TJA was recorded, the raters used a previously published form to score technique flaws for each participant^46^. Each of the 10 items was scored as 1 (no flaw) or 0 (flaw). The items of TJA were as follows:

1. Lower extremity valgus at landing;
2. Thighs do not reach parallel;
3. Thighs not equal side-to-side;
4. Foot placement not shoulder width apart;
5. Foot placement not parallel;
6. Foot contact timing not equal;
7. Excessive landing contact noise;
8. Pause between jumps;
9. Technique declines prior to 10 s;
10. Does not land on same footprint.

The reported intra-tester and inter-tester reliability for the TJA test was very good–excellent. Average percentage of exact agreement (PEA) between the 2 testers across all scoring criteria for all subjects was 93% (range 80–100%). The kappa measure of agreement was k = 0.88. The intra-tester PEA ranged from 87.2 to 100%, with kappa values of k = 0.86–1.0^47^.

### Statistical analysis

Statistical analysis was performed using STATISTICA 12.0 Pl software. The Shapiro–Wilk test was conducted to assess normality of data. The two-way mixed ANOVA with 3 groups (between-subjects effect) and 2 limbs (within-subjects effect) was used to determine the significance of differences regarding the Y-balance test. When a significant main effect was detected, Tukey’s post-hoc test was applied. The Kruskal–Wallis nonparametric test was used to determine the differences between groups in FMS and TJA tests. When a significant main effect was detected, a non-parametric post-hoc test was performed. The effect size was calculated using Cohen’s *d* and interpreted as small (0.2–0.3), medium (0.5) or large (> 0.8). Differences were considered statistically significant at the level of (*p* < 0.05).

## Results

### Functional movement screen test

There were no significant differences between groups in any of the 7 FMS tasks. The ACL group had only significantly lower composite score compared to subjects from the C group (12 ± 4 vs. 15 ± 2) (Table 2). Also, in the MI group the composite score was higher than in the ACL group, but the difference was non-significant (14 ± 3 vs. 12 ± 4) (Table 2).

**Table 2:** Comparison of Functional Movement Screen variables between study groups.

Outcome measure	Side	ACL group	*p*#	MI group 2	*p*#	C group 3	*p*#	p*	p**	p***	H
Deep squat		1 ± 1 (1–2)		2 ± 0 (2–2)		2 ± 1 (1–2)		0.274	0310	0.360	4.36
Hurdle step	I	2 ± 0 (2–2)	0.695	2 ± 0 (2–2)	0.531	2 ± 0 (2–2)	0.409	0.626	0.771	0.170	1.59
	U	2 ± 0 (2–2)		2 ± 0 (2–2)		2 ± 1 (2–3)		0.363	0.933	0.837	3.03
In-line lunge	I	2 ± 0 (2–2)	0.576	2 ± 0 (2–2)	0.987	2 ± 0 (2–2)	0.746	0.331	0413	0.331	0.23
	U	2 ± 0 (2–2)		2 ± 0 (2–2)		2 ± 1 (2–3)		0.446	0.759	0.654	2.75
Shoulder mobility	I	3 ± 1 (2–3)	0.907	3 ± 0 (3–3)	0.832	3 ± 0 (3–3)	0.751	0.557	0.313	0.881	1.59
	U	3 ± 0 (3–3)		3 ± 0 (3–3)		3 ± 0 (3–3)		0.400	0.783	0.679	4.67
Active straight leg raise	I	2 ± 0 (2–2)	0.410	2 ± 0 (2–2)	0.649	2 ± 0 (2–2)	0.861	0.285	0.663	0.656	0.82
	U	2 ± 1 (1–2)		2 ± 0 (2–2)		2 ± 1 (2–3)		0.928	0.642	0.811	6.10
Trunk stability push up		2 ± 1 (2–3)		3 ± 1 (2–3)		2 ± 1 (2–3)		0.410	0.172	0.571	3.16
Rotary stability	I	2 ± 0 (2–2)	0.549	2 ± 0 (2–2)	0.713	2 ± 0 (2–2)	0.549	0.773	0.237	0.925	4.63
	U	2 ± 0 (2–2)		2 ± 0 (2–2)		2 ± 0 (2–2)		0.659	0.240	0.413	0.98
Composite score		12 ± 4 (11–15)		14 ± 3 (13–16)		15 ± 2 (14–16)		0.068	0.739	**0.007**	10.41

Values are expressed as median ± quantile range (lower quantile– upper quantile) of points from FMS test.

H, Kruskal–Wallis test value between study groups.

p* *p* value between ACL group and MI group (the *p* value there is the post-hoc of study groups main effect).

p** *p* value between MI group and C group (the *p* value there is the post-hoc of study groups main effect).

p*** *p* value between ACL group and C group (the *p* value there is the post-hoc of study groups main effect).

*p#* *p* value between involved (I) and uninvolved (U) side within each group.

### Y-balance test

The subjects in the ACL group demonstrated lower values for all 3 reaching directions as well as composite score compared to the remaining 2 groups. However, differences were significant only for anterior, posterior-lateral reach and composite score. In anterior direction, the ACL group demonstrated significantly lower reach distances for the injured leg in comparison to the MI group and C group (69.2 ± 5.7 vs. 74.8 ± 4.5 vs. 74.0 ± 5.6). Furthermore, in the case of the uninjured leg, anterior reach distance was significantly lower in the ACL group compared to the MI group (70.0 ± 5.6 vs. 73.7 ± 3.9) (Table 3). In the posterior-lateral direction, reach distance for the uninjured leg was significantly lower in the ACL group compared to the C group (103.2 ± 6.4 vs. 108.5 ± 6.0) (Table 3). Composite score for the injured leg demonstrated significantly lower values in the ACL group compared to the MI group (93.9 ± 4.4 vs. 97.9 ± 4.3). The difference between the ACL and C groups was non-significant, however, the effect size was high. Composite score for the uninjured leg was significantly lower in the ACL group compared to the C group (94.5 ± 4.4 vs. 97.7 ± 4.8). T here were no significant asymmetries for any of the reach directions in the evaluated groups (Table 3). None significant group by limbs interaction was found.

**Table 3:** Comparison of Y-balance variables between study groups.

Outcome measure	Side	ACL group	*p*#	MI group	*p#*	C group	*p#*	p*	ES	p**	ES	p***	ES
Anterior reach (%)	I	69.2 ± 5.7	0.952	74.8 ± 4.5	0.760	74.0 ± 5.6	0.181	**0.003**	1.09	0.994	0.15	**0.01**	0.84
	U	70.0 ± 5.6		73.7 ± 3.9		73.1 ± 5.8		**0.03**	0.76	0.998	0.12	0.432	0.54
Posterolateral reach (%)	I	103.3 ± 6.1	0.965	107.6 ± 7.1	0.552	107.1 ± 6.8	0.130	0.309	0.64	0.999	0.07	0.435	0.58
	U	103.2 ± 6.4		106.8 ± 7.7		108.5 ± 6.0		0.539	0.50	0.958	0.24	**0.03**	0.85
Posteromedial reach (%)	I	109.2 ± 5.2	0.920	111.3 ± 5.3	0.958	110.7 ± 6.1	0.789	0.826	0.39	0.999	0.10	0.949	0.26
	U	110.1 ± 4.9		110.6 ± 5.0		111.4 ± 5.8		0.999	0.10	0.997	0.14	0.972	0.24
Composite score (%)	I	93.9 ± 4.4	0.971	97.9 ± 4.3	0.256	97.3 ± 5.2	0.104	**0.02**	0.91	0.997	0.12	0.185	0.70
	U	94.5 ± 4.4		97.0 ± 4.4		97.7 ± 4.8		0.472	0.56	0.997	0.15	**0.03**	0.69

*p* < 0.05 values are indicated in bold.

Values are expressed as Mean ± SD.

H, Kruskal–Wallis test value between study groups.

ES, effect size (Cohen d) between study groups.

p* *p* value between ACL group and MI group (the *p* value there is the post-hoc of study groups main effect).

p** *p* value between MI group and C group (the *p* value there is the post-hoc of study groups main effect).

p*** *p* value between ACL group and C group (the *p* value there is the post-hoc of study groups main effect).

*p*# *p* value between involved (I) and uninvolved (U) side within each group.

### Tuck jump assessment test

The ACL group demonstrated lower scores for the TJA test in comparison to the MI group for items 2, 3 and 9. The values were significantly lower in the ACL group compared to the C group for items 1 and 2 (Table 4). Considering item 4, significant differences were noted between the ACL, MI and C groups, but subjects following ACL reconstruction demonstrated higher values (Table 4). There were no significant differences between groups in TJA composite score (Table 4).

**Table. 4:** Comparison of Tuck Jump Assessment variables between study groups.

Outcome measure	ACL group	p*	MI group	p**	C group	p***	H
1. Lower extremity valgus at landing	0 ± 1 (0–1)	0.140	0 ± 0 (0–0)	0.310	0 ± 0 (0–0)	**0.018**	6.36
2. Thighs do not reach parallel	1 ± 1 (0–1)	**0.016**	1 ± 0 (1–1)	0.572	1 ± 0 (1–1)	**0.004**	11.48
3. Thighs not equal side-to-side	0 ± 0 (0–0)	**0.028**	0 ± 0 (0–0)	0.696	0 ± 0 (0–0)	0.061	4.65
4. Foot placement not shoulder width apart	1 ± 0 (1–1)	**0.004**	0 ± 1 (0–1)	0.359	0 ± 1 (0–1)	**0.0002**	14.48
5. Foot placement not parallel	1 ± 1 (0–1)	0.350	1 ± 1 (0–1)	0.074	1 ± 0 (1–1)	0.242	4.12
6. Foot contact timing not equal	0 ± 0 (0–0)	0.582	0 ± 0 (0–0)	0.085	0 ± 1 (0–1)	0.212	3.42
7. Excessive landing contact noise	1 ± 0 (1–1)	0.305	1 ± 0 (1–1)	0.985	1 ± 0 (1–1)	0.305	1.34
8. Pause between jumps	1 ± 1 (0–1)	0.800	1 ± 1 (0–1)	0.484	1 ± 0 (1–1)	0.646	0.52
9. Technique declines prior to 10 s	0 ± 0 (0–0)	**0.027**	0 ± 1 (0–1)	0.770	0 ± 1 (0–1)	0.061	5.51
10. Does not land in same footprint	0 ± 0 (0–0)	0.069	0 ± 0 (0–0)	0.080	0 ± 0 (0–0)	0.071	6.63
Composite score	5 ± 1 (4–5)	0.741	5 ± 1 (4–5)	0.523	5 ± 2 (4–6)	0.561	1.03

*p* < 0.05 values are indicated in bold.

Values are expressed as median ± quantile range (lower quantile − upper quantile) of points from TJA test.

H, Kruskal–Wallis test value between study groups.

p* *p* value between ACL group and C group (the *p* value there is the post-hoc of study groups main effect).

p** *p* value between MI group and C group (the *p* value there is the post-hoc of study groups main effect).

p*** *p* value between ACL group and C group (the *p* value there is the post-hoc of study groups main effect).

## Discussion

The most important information in this study is that athletes following ACL reconstruction still have deficits in movement patterns or in neuromuscular control despite being cleared for sport performance and passing the RTS. The Y-balance and TJA tests are useful assessment methods regarding these impairments.

In this study, the subjects after ACL reconstruction demonstrated lower values in all 3 reaching directions as well as in composite score compared to the remaining two groups. However, these differences were significant only in the case of anterior, posterior-lateral reach and composite score. What is of significance in this study is that the performance of the FMS test was similar in all three groups of football players, independent of their injury history. Only the composite score was significantly lower among subjects after ACL reconstruction compared to the uninjured control athletes. Furthermore, subjects following ACL reconstruction demonstrated lower performance values for the TJA test compared to the remaining groups.

It has been demonstrated that low FMS composite scores (≤ 14) may be associated with increased risk of injury while also predicting trauma^39–41^. The results obtained in the study by Shojaedin et al.^48^ indicated that athletes with pre-season FMS scores less than 17 were 4.7 times more likely to sustain lower limb injuries. Moreover, in some studies, the FMS has been implemented as one of the assessment tools used during RTS evaluation^14,18^. Mayer et al.^8^ did not observe any differences in FMS composite scores between subjects following injury who were cleared for return to sport without restrictions and those who were not. Scores for both groups were below 14, which was reported as the injury predictive threshold. They suggested that all evaluated subjects were deficient in neuromuscular control and, therefore, at an increased risk of injury regardless of whether they were cleared for return to sport or not^8^. Thus, it seems that passing the RTS criteria does not mean that return to play is safe or that all the consequences of the injury are healed. Chimera et al.^14^ have also suggested that FMS composite score can be similar between injured and uninjured individuals, but these scores may be obtained with different individual movement patterns. These results are similar to the authors’ observations, in which no significant differences between groups in any of the 7 FMS tasks were noted. Only the composite score was significantly lower for subjects after ACL reconstruction compared to the uninjured control group.

Many researchers have noted a relationship between dynamic balance performance and injury risk^16,20,30^. Clagg et al.^32^ compared performance using the Y-balance test between participants after ACL reconstruction at the time of return to sport and uninjured subjects. The ACL group showed worse anterior reach performance for both the involved and uninvolved limbs compared to the control group, but there were no differences in the posterior-medial or posterior-lateral reach directions or in limb symmetry indices. In other studies, decreased anterior reach and composite scores have also been exhibited^16,20,49^. Similar results were noted in this study, where subjects after ACL reconstruction demonstrated significantly worse anterior and posterior-lateral reach performance in comparison to the remaining two groups. But what is important, Clagg et al.^32^ evaluated athletes at the time of return to sport, in the authors’ study, similar deficits in dynamic control were observed later, when they were cleared for sport performance and passed the RTS, actively participating in the game. Therefore, the authors have hypothesized that anterior and posterior-medial reach performance of the Y-balance test may be sensitive, detecting specific deficiencies in dynamic balance due to ACL functional deficiency.

The hamstring muscles act as agonists to the ACL by resisting anterior tibial displacement that results from quadriceps muscle forces in the knee^12^. Therefore, decreased hamstring muscle strength has been suggested as a risk factor for ACL rupture^12,50^. As was reported by Kim et al.^51^, in patients with ACL, tear decreases were observed in both the quadriceps and hamstring muscles. Weakness of hamstring muscles and insufficiency in resisting anterior tibial displacement after ACL rupture may explain the significantly lower anterior reach distance in both the affected and unaffected limbs observed in this work and in studies by other authors^52^. As was previously reported, hamstring muscle weakness is strongly related to gastrocnemius muscle hyperactivity and a following decrease in ankle dorsiflexion, which may be a probable reason for the reported lower reach distance in the Y-balance test^53,54^.

Mayer et al.^8^ did not observe difference between Y-balance test composite score and individual reach direction distances between athletes after ACL reconstruction who were cleared to return to sport and those who were not. As they underlined, in both groups, composite score was below 94.0%, which had been previously linked with elevated risk of injury^8^. They concluded that in these athletes, movement deficits may have existed before the initial ACL rupture and thus, may be associated with initial injury. In this study, only the group following ACL reconstruction had a composite score below 94% for both limbs. In contrast, the group after minor injuries and the non-injured controls had composite scores above this value. This may suggest that such severe traumas as ACL rupture and reconstruction leave much greater deficiencies in dynamic control than other types of injuries. This also indicates that deficits in anterior reach distance are specific for people after ACL reconstruction and still occur despite passing other RTS tests. Therefore, without any targeted assessment and treatment of these specific deficits, the risk of ACL re-injury may be considered permanent.

The TJA is a plyometric test, identifying jumping and landing technique flaws, which aims to identify five risk factors associated with ACL injury, such as ligament dominance, quadriceps dominance, leg dominance, trunk dominance and technique perfection^21,23^. Ligament dominance is described as imbalance between the neuromuscular and ligamentous control of dynamic knee stability. Quadriceps dominance is imbalance between knee extensor and flexor strength, recruitment and coordination. Leg dominance is treated as imbalance between both lower limbs in strength, coordination and control. Trunk dominance concerns imbalance of core control and the coordination for its resistance during movement^37,46,55^. The TJA protocol requires athletes to perform consecutive jumps for 10 s, therefore, it is used to evaluate the potential fatigue effect that may extenuate landing flaws. In the authors’ study, subjects after ACL reconstruction demonstrated lower performance for items 1, 2, 3 and 9 of TJA compared to the remaining groups. Item 1 mainly regards the ligament dominance risk factor. Item 2 is related to the quadriceps dominance risk factor, which describes imbalance between the quadriceps and hamstring muscles. This imbalance was also noted during the Y-balance test as decreased anterior reach distance. Also, the leg dominance risk factor was higher in the ACL group, which was noted as item 3. The fatigue effect was seen in item 9 as a decline in jump technique.

It should be noted that during the TJA test, fatigue may influence landing flaws and therefore, this test may more accurately reflect the biomechanical conditions which occur during the game. This factor does not appear in the FMS or Y-balance tests, both performed in non-fatigued conditions. On the other hand, it has been reported that non-sport-specific bilateral jumping tasks in TJA are generic and not related to ACL injury, because non-contact ACL injuries occur almost exclusively during unilateral landing^23^. But despite its generality, the use of TJA to identify neuromuscular imbalances may provide direction for targeted treatment among athletes following ACL reconstruction^22^. Therefore, in active football players cleared to play sport, deficits in neuromuscular control may be assessed and monitored with repeated measurements using TJA.

Analyzing the effectiveness of the FMS, Y-balance and TJA tests in the assessment of players cleared to play after ACL reconstruction, it should be noted that only selected elements of the Y-balance and TJA tests were sensitive to deficits in dynamic balance and neuromuscular control existing after ACL reconstruction, despite passing the RTS process by these players. The significant differences in these parameters between players following ACL reconstruction and players after minor injuries and those without injuries indicate that such serious traumas as ACL rupture and reconstruction permanently disrupt movement patterns and neuromuscular control. Therefore, athletes cleared to play after ACL reconstruction should be continuously monitored to diminish the negative impact of remaining motor deficits which may potentially lead to re-injury. It is also important to use appropriate tests and tools for this monitoring. These test should be able to distinguish ACL-related deficits from those that are a general problem of mobility and stability commonly found in athletes.

There are some limitations which should be addressed. The study design was observational and the football players were evaluated only once. Therefore, we cannot observe the real cause-effect relationship between the volume of neuromuscular deficits and their influence on the ACL re-injury risk, which would be possible via longitudinal studies. There is a need for future research among a large group of athletes after ACL reconstruction, including longitudinal monitoring of factors related to ACL re-injury.

## Conclusions

The authors conclude that athletes following ACL reconstruction still have deficits in movement patterns or in neuromuscular control despite passing RTS and being cleared to play sport. Creating a set of sufficiently sensitive assessment methods may significantly reduce the potential risk of injury due to the fact that diagnosed and monitored deficits may be treated on an ongoing basis. The authors suggest that individual elements of the Y-balance and TJA tests may be suitable for such specific assessment.

## Data Availability

All data generated or analyzed during this study are included in this published article.
